# Measuring the Psychological Security of Urban Residents: Construction and Validation of a New Scale

**DOI:** 10.3389/fpsyg.2019.02423

**Published:** 2019-10-25

**Authors:** Jiaqi Wang, Ruyin Long, Hong Chen, Qianwen Li

**Affiliations:** ^1^School of Management, China University of Mining and Technology, Xuzhou, China; ^2^Research Center for Energy Economics, School of Business Administration, Henan Polytechnic University, Jiaozuo, China

**Keywords:** psychological security, urban residents, scale development, grounded theory, quantitative analysis

## Abstract

With the acceleration of urbanization in developing countries, resources relating to medical care and the environment are becoming increasingly scarce, and the negative spillover effects brought about by scientific and technological progress have also significantly increased the pressure on urban residents. The psychological security of urban residents has recently undergone significant change. This paper introduces psychological security into the area of urban residents’ lives, defines the concept of urban residents’ psychological security, and presents the development and validation of the Urban Residents Psychological Security Scale (URPS). By considering psychological indicators, this paper supplements our knowledge on environmental indicators such as the risk perception of environmental pollution and climate change, and social indicators such as urban belongingness and the risk perception of technology which verifies the negative spillover effects of technological development. Based on a literature search and consideration of grounded theory (25 urban residents’ in-depth interview records), the psychological security of urban residents is divided into three dimensions: self-psychological security, social environmental security, and natural environmental security, consisting of 20 items. In this study, 802 questionnaires were completed by participants. We determined that the URPS scale has good reliability and validity using exploratory factor analysis and confirmatory factor analysis, and conclude that the scale can be used as an effective measurement tool for urban residents’ psychological security. The development of this scale has important theoretical and practical significance in helping city managers better understand the residents’ demands and to monitor the implementation effects of policies.

## Introduction

With the acceleration of economic development and urbanization in developing countries, profound changes have occurred in aspects such as the economic system, social structure, and values. These changes modified people’s original ways of thinking and even their lifestyles. The inherent requirements for people in relation to quality of life and environmental safety are rapidly coming to developed countries. In addition, studies increasingly show that air pollution, soil pollution, climate change, and so on, will not only affect people’s physical health (Burnett et al., 2018; Zhang et al., 2018) but also indirectly or directly harm people’s mental health (Evans, 2003; Chen et al., 2018; Obradovich et al., 2018). The continuous advancement of technologies such as the Internet and artificial intelligence has a significantly positive impact on remotely connecting relationships and increasing productivity but can also lead to negative effects such as unwanted personal information disclosure, Internet addiction, and social anxiety (Chesley, 2005; Gámez-Guadix and Calvete, 2016; Jia et al., 2017). Few researchers have systematically studied the negative spillover effects of technological progress, and even fewer have incorporated these effects into psychology.

As a decisive factor of mental health, psychological security has been widely concerned. Maslow defined psychological security as “a feeling of confidence, safety and freedom that separates from fear and anxiety, and especially the feeling of satisfying one’s needs now (and in the future).”

In previous studies, most research on psychological security has focused on the workplace (Probst, 2002; Hu et al., 2018), and the psychological security of urban residents has not received sufficient attention. The psychological security of urban residents has mainly consisted of fear of crime, public security or social security, most of which are directly related to social factors such as public security, food safety, and medical supervision. However, insecurity is shaped by everyday experiences and often is more related to experiences of living in a risky society than to only criminal incidents (Garland, 2000). Therefore, the psychological security of urban residents should be a complex multidimensional structure rather than a simple one-dimensional structure. By analyzing and summarizing the literature, the psychological security of urban residents can be divided into three categories: psychological, social, and environmental. Most studies have focused only on the influences of individual psychological factors and social factors (Edmondson and Lei, 2014; Oishi and Kesebir, 2015; Soto, 2015), whereas insufficient attention has been paid to the effects of environmental factors. The traditional structural dimension cannot adapt to actual needs, and there is not currently available a scale that matches the actuality.

**FIGURE 1:**
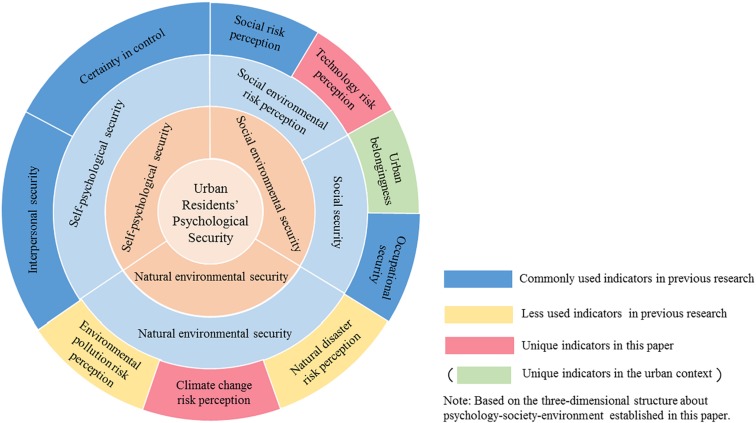
The model of urban residents’ psychological security.

On the basis of the arguments developed above, we summarized the concept of residents’ psychological security at the city level by combining them with the practical needs, explored and developed the Urban Residents’ Psychological Security Scale (URPS) scale to be applicable to the current environment, and verified the applicability of the three-dimensional structure including psychological, social and environmental factors. As shown in Figure 1, on the basis of traditional indicators, such as interpersonal security, certainty in control, social risk perception, and occupational security, we incorporated environmental pollution risk perception and natural disaster risk perception into the structure of urban residents’ psychological security, while considering the indicators of technology risk perception, urban belongingness and climate change risk perception. Among the indicators, urban belongingness was a unique indicator of psychological security at the city level.

The URPS scale developed in this paper could help city managers understand the security status of urban residents, including psychological, social, and environmental aspects, and could help relevant departments formulate targeted intervention policies. In the future, this scale is expected to effectively enhance urban attractiveness, improve the urban integration of migrants and reduce the crime rate.

The remainder of this paper is arranged as follows. Section “Literature Review” describes the related research on the psychological security of urban residents. The qualitative analysis method of Grounded Theory is used to construct the initial scale of psychological security of urban residents in section “Initial Scale Construction Based on Grounded Theory.” In section “Quantitative Method,” we purify the scale and test its reliability and validity using data from pre-survey and formal survey. The results of this study are discussed further in section “Discussion and Conclusion” and the conclusions are given. The section “Limitations and Future Studies” is the limitations of this study and directions for future research.

## Literature Review

### Concept and Dimensions

Cong and An (2004) defined psychological security according to Maslow (1942) as the presentiment that may arise from dangers or risks in the physiology or the psychology of the individual, as well as the sense of powerfulness and powerlessness of the individual in dealing with dangers or risks, mainly related to the sense of certainty and controllability. It is widely used by researchers (Sun and Yao, 2009; Zhao and Jing, 2013; Yu and Zhao, 2016). Hart et al. (2005) and Hart (2014) believes that psychological insecurities refer to each individual’s anxiety about potential harm and threat. Obviously, the sense of psychological security is a subjective judgment of whether the individual’s environment is deterministic and controllable, and the state of consciousness based on his or her own personality traits.

According to the above literature, the characteristics of psychological security can be summarized as follows: (1) psychological security is an emotional experience perceived by the individual. This emotional experience is derived from external stimuli and is determined by both the intensity of the stimulus and the psychological quality of the individual. (2) The expression of psychological security is mainly the certainty, control, and risk premonition felt by the individual. (3) Psychological security will affect physical and mental health. Individuals with higher psychological security will experience more confidence and freedom while individuals with lower psychological security are more prone to anxiety or fear, and even depression. Differences in the personality and environmental perception of individuals determine the level of the individual’s trust in the outside world, and is self-centered and based on the objective environment. Individuals then further evaluate and decide whether or not the outside world is safe, and that usually connects with the degree of recognition with the outside world or the degree of willingness to contribute to it. Therefore, the connotations of individual psychological security change with the environmental background, for example, individual psychological security in the workplace. Carmeli and Gittell (2009) effectively combined personal perceptions in the social and work fields, and believed that psychological security refers to people’s views on their social environment and work environment, as well as their perceived reactions to risk-taking behaviors in the workplace. By combining individuals’ perceptions of themselves, society and the urban environment, we attempted to introduce psychological security into the background of urban life, and we defined urban residents’ psychological security as the risk judgment of individuals living in cities of their own urban living conditions based on past experience or intuition.

All human emotions are derived from the direct feelings of the heart. The certainty in control is one of the important and widely used dimensions of psychological security (Zhao and Jing, 2013; Yu and Zhao, 2016). Loss of control not only changes the individual’s perceptions, beliefs, and behaviors but also affects their physical and mental health (Whitson and Galinsky, 2008). At the same time, individuals in the city will also have various types of interpersonal needs in their social lives. Demir (2008), Edmondson and Lei (2014), and Inoue et al. (2016) found that there is a significant correlation between interpersonal relationships and the sense of security. Safe and supportive social relationships are not only beneficial to individuals (Kagan, 2009) but also promote prosocial behavior (Mikulincer and Shaver, 2007). Negative interpersonal events can cause individuals to feel anxiety and other similar emotions while positive interpersonal experiences will effectively reduce attachment anxiety (Davila and Sargent, 2003; Zhang, 2009). Individuals with higher levels of interpersonal trust and interpersonal security will perceive fewer negative events and thus have a higher sense of psychological security.

The psychological security of residents is also affected by external objective factors. In addition to the economic development of the city, the key factors determining whether the local residents leave and whether foreigners stay for a long time are people’s familiarity with the urban environment and the degree of recognition with the urban atmosphere. As well as the economic level of the city, the key factors determining whether the local residents leave and whether transient populations stay for a long time are people’s familiarity with the urban area and the degree of recognition with the urban atmosphere. This emotional element is known as urban belongingness, a unique indicator of psychological security in the urban context. The individual’s demand for belongingness is due to the desire for security. The need for a sense of belonging stems from the desire for security. Factors such as equity protection, housing status, and social integration will reduce the sense of belongingness and the urban identity of the non-native population who work and live in the city, which will result in their relatively isolated social relationships, cultural activities, and political participation, thus affecting the city’s social and economic development. Economic factors also determine the psychological security of urban residents to a certain extent (Van Hal, 2015), which is reflected in occupational stability and occupational risk. In addition, a large number of studies have shown that the fear of crime in terms of social security factors will increase people’s psychological pressure (Astell-Burt et al., 2015), and have a negative impact on their sense of security and well-being (Foster et al., 2016; Prieto Curiel and Bishop, 2017). Carter et al. (2011), Ross and Hill (2013), and Tseng et al. (2017) have also found such negative effects from food insecurity.

In recent years, the emergence of natural disasters and environmental pollution has caused people to frequently feel a sense of having lost control. Publicity and education on energy conservation, emission reduction, and green and low carbons have made more people aware of the urgency of environmental protection issues. Doherty and Clayton (2011) found that climate change threatens the emotional health of people by making them worry or feel uncertain about future risks. The haze affects the psychological and physical health of people who live in a polluted area. The perception of smog risk even leads to the outflow of talents in smog-polluted areas (Lu and Long, 2018). Sekulova and Van den Bergh (2016) argue that natural disasters, which may be considered to be large-scale traumatic events, not only cause considerable material losses, but also can seriously impair psychological health. The challenge of tackling climate change and environmental pollution has become increasingly critical, and a series of social surveys are needed to improve the ability of psychologists and governments to cope with the relevant impacts of this.

In conclusion, the psychological security of urban residents is the risk judgment of individuals living in cities for their own state and urban living conditions based on past experience or intuition. The dimensions include (1) self-psychological security, that is, the individual’s safety expectations for future life based on past life experiences, and their positive experiences of maintaining a favorable position in their own situation through the process of interpersonal interaction. (2) Social environmental security, reflecting residents’ psychological attachment and identity with the city they live in, and their comprehensive risk perception of their social environment, urban atmosphere, and professional status. (3) Natural environmental security, that is, the risk perception of urban residents toward their living urban natural environment.

### Measurement

Some representative results of psychological security dimension and scale research are shown in Table 1. At present, there are few researchers paying attention to the measurement of the psychological security of urban residents. Most research is focused on measuring psychological safety in the workplace, in which individual-level studies of employees are mostly assessed using the Dyadic Psychological Safety Items designed by Tynan (2005). This scale includes two dimensions of self-psychological safety and other-psychological safety, with a total of 12 items. Team-level studies are mostly conducted using the Team Psychological Safety Scale (Edmondson, 1999). The scale contains seven self-evaluation items, and there are no separate dimensions. Most researchers have used a revised version of this scale (Pearsall and Ellis, 2011; Leroy et al., 2012; Hood et al., 2016). The Psychological Climate Scale developed by Brown and Leigh (1996) is widely used in organizational-level studies (Ogilvie et al., 2017; Ho et al., 2018) and includes measurement of supportive management, role clarity, contribution, recognition, self-expression, and challenges, consisting of 21 items. However, the dimension setting is applicable to only occupational sites but not to urban residents. Zani et al.’s, (2001) research on adolescents had similar problems.

**TABLE 1:** Psychological security dimension.

**Study**	**Dimension**	**Research object**
Maslow, 1942	Safety, belongingness and receiving love and affection	–
Brown and Leigh, 1996	Supportive management, role clarity, contribution, recognition, self-expression and challenge	Employee
Edmondson, 1999	No dimension	Employee
Zani et al., 2001	Cognitive (perceived seriousness of problems in living environment), emotional (worried degree of negative events) and behavioral (behaviors to face feeling of unsafety)	Adolescent
Cong and An, 2004	Interpersonal security and certainty in control	–
Tynan, 2005	Self-psychological security and other psychological security	Employee
Dzhamalova et al., 2016	Senses and feelings, perception and evaluation of reality according to the criterion of dangerous-safe, and analysis and forecasting for a secure future	–
Yin, 1980	Risk estimation and severity evaluation of injury	Resident
Van der Wurff et al., 1989	Attractivity for crime, evil intent and power (feelings of self-assurance, control and confidence in meeting crime) and criminalizable space	Resident
Hale, 1996	Street crime, emotional security, physical security and property security	Resident
Vail, 1999	Property security, personal security, traffic security, medical security, food security and labor security	Resident
Rader, 2004	Emotive component (fear of crime), cognitive component (perceived risk), and behavioral component (constrained behaviors)	Resident
Xia and Wei, 2011	Economic security, interpersonal security, social security, environmental security, and survival security	Resident

Maslow (1942) developed the Psychological Security-Insecurity Questionnaire and believed that psychological security can be divided into three dimensions: safety; belongingness; and receiving love and affection. The Security Questionnaire developed by Cong and An (2004) includes two dimensions: interpersonal security and certainty in control. Both measurement tools and dimensions are widely used, but because the research subjects are not limited, the questionnaire must be adapted to specific situations. In recent years, some researchers have incorporated the perception of social reality into the structure of psychological security. For example, on the basis of the external perception of stable personality, Dzhamalova et al. (2016) believe that psychological security consists of senses and feelings, perception and evaluation of reality according to the dangerous-safe criterion, and analysis and forecasting for a secure future. The psychological security state at the city level is based on the individual psychological state and is influenced by environmental factors. Previous studies have mainly focused on the fear of crime (Yin, 1980; Van der Wurff et al., 1989; Rader, 2004). Hale (1996) combined emotional and social factors to divide psychological security into four dimensions: street crime, emotional security, physical security, and property security. Vail (1999) considered more social factors and constructed a six-dimensional structure including property security, personal security, traffic security, medical security, food security, and labor security. The Resident’s Sense of Security Scale developed by Xia and Wei (2011) includes factors of economic security, interpersonal security, social security, environmental security, and survival security. This research incorporates elements from psychological factors, social factors, and environmental factors, but it is not comprehensive.

The previous scales measuring psychological security mostly focus on the multi-level security of the employees in the workplace. Other than this, the research focus of other scales has been diverse but scattered, and the degree of recognition is generally not high, and application field and scene are limited. A specific questionnaire to measure urban residents’ psychological security is lacking. Therefore, it is important to develop a scale of urban residents’ psychological security based on three-dimensional structure of psychology, society, and environment, which reflects social reality.

Thus, the connotation of psychological security of urban residents has changed over time, and the existing literature is lacking in terms of reflecting the comprehensive indicators of psychological, social, and environmental aspects. The development of the URPS scale has expanded the work in this field to some extent. Moreover, the grounded theory emphasizes the utilization of original data and fills the gap between theory and reality through methods such as literature review, interviewing, and coding, which can effectively address the defects in previous research in this field (Glaser et al., 1968). Consequently, we used a combination of qualitative and quantitative methods to develop the URPS scale, based on extensive literature research. We used the grounded theory to develop the initial scale and used the data collected through investigation questionnaires to quantitatively analyze the structure of the URPS scale.

## Initial Scale Construction Based on Grounded Theory

### Participants and Design

In order to extract the items for the initial UPRS scale, we conceptualized urban residents’ psychological security and presented the specific performances of its structure. We obtained the original items using the following methods: (1) we conducted targeted interviews of urban residents and used recording software to reorganize, edit, and export the interviews. (2) We reviewed the existing literature and systematically analyzed the theory and empirical research results regarding security and psychological security to provide theoretical support for the scale.

The interviews did not include pre-set patterns or pre-assumptions but did consist of a specific outline. The outline was an auxiliary tool for us to guide interviewees by reviewing and describing relevant question, which is provided in Table 2 below.

**TABLE 2:** Outline of interview on psychological security of urban residents.

**Theme**	**Main content**
Basic information	Gender, age, educational background, monthly income level, work place, nature of organization
The status of urban residents’ psychological security	a. What do you think of the city you live in now? How does it feel to live in this city? b. What advantages do you think this city has? What are the shortcomings? c. Do you sometimes feel worried, anxious, panic or afraid in your daily life in this city? Can you give me an example?
The structure of urban residents’ psychological security	a. Can you describe the situation and feelings when you feel safe in your daily life? b. Can you describe the situation and feelings when you feel safe at work?

The questions listed in Table 2 are only for reference. The interview was adjusted according to each specific situation. In addition to obtaining basic information, we conducted extended interviews depending on the interviewee reactions or answers.

### Ethics Statement

This study was carried out in accordance with the principles of the Basel Declaration and recommendations of Ethical Codes of Consulting and Clinical Psychology of Chinese Psychological Society, Chinese Psychological Society. The protocol was approved by the Ethics Committee at the Department of Organizational and Behavioral Sciences, China University of Mining and Technology. All subjects gave written informed consent in accordance with the Declaration of Helsinki. Before the interview, the interviewees were told that they would be recorded and that we would fully respect their wishes.

### Procedure

Based on the grounded theory and research requirements, we needed urban residents as the research subjects, with different educational backgrounds, different income levels, and mainly young and middle-aged people. Therefore, 25 interviewees were randomly selected through online recruitment. We conducted descriptive statistical analysis on the basic information of the interviewees. The results indicated that 52% were males, and 48% were females; 36% were between ages 22 and 30, 32% were between 31 and 40, and 32% were over 40; and 68% had received undergraduate education or above. In addition, our study included urban residents with different income levels and city. The sample is representative.

We converted the interview recordings into text, and on completion had obtained interview records of about 30,000 words. Eight respondents were randomly selected for theoretical saturation test, but their answers did not bring new information to the research, that is, the content was saturated in theory. The researchers read the original text content of the interview word by word, collected phrases about psychological security, and extracted conceptual labels from them. In order to ensure the objectivity of the label, the extracted statements were the original words of the interviewee.

After preliminary classification, 22 items were obtained from a total of 133 original statements. The researchers discussed the statements several times and decided to reclassify them according to their semantic similarity and delete ambiguous items, meaning that 126 statements remained. Due to the complexity of the 126 statements, the researchers combined and simplified them based on the literature review to form conceptual indicators. The specific classification is shown in Table 3.

**TABLE 3:** Classification of the semantically similar items.

**Original statements in the interview text**	**Conceptualization**	**Frequency**
The environment is not very good, food is not safe, the network is not safe, and it is easy to be scammed by the Internet, all of these make me feel unsafe that living in this city; In foreign countries, people can carry guns and dare not go out in the middle of the night. I think China has done a very good job in this regard and I hope to increase the sanctions on robbery and theft; I am afraid to eat gutter oil, food is not safe, there are many pesticides and fertilizers	Social risk perception	25
I work too long every day and worry about my health; My job is very stable, my income is very guaranteed, and these are my sense of security; Large work pressure, fast life pace, I am busy every day. If I am not at work, I am on the way to work.	Occupational security	20
I am not an egoist, and I will get happiness by helping others; There is not much intimacy between people, everyone is very busy, and they don’t want to take care of you; There are a few friends in my daily life. When I have a job, I can have a dinner party or go out to play with my colleagues	Interpersonal security	15
After graduating, I moved around several places and eventually returned to my hometown. To be honest, the economy here is not particularly developed, and the job opportunities are relatively small, but ultimately it is my hometown; The pace of life in this city is not fast, prices are not low, and the environment is not good. The reason why I stay here is that my family is here; This city is my home. No matter how long I play outside, I still have to come back	Urban belongingness	15
The air quality is not good, and the haze will pollute various things and damage the health; The haze is frequent, and the environment is relatively poor. Now, the city managers are trying to improve the situation, I hope that we can persevere to manage the environment, which is a benefit for the local people and the outsiders; Concerned about air quality, pollution problems in heavy industrial cities are very serious	Environmental pollution risk perception	14
Surrounding people live according to the established model, without any incitement or turmoil; Being able to have a safe environment to ensure that I can implement my plan and I will not interrupt my plan or fail to complete it due to some unexpected circumstances; I am worried that housing prices will become higher, I am anxious about the growth rate of wages, and I am afraid of the development of IT industry in Xuzhou. I am scared that 1 day I need to leave my hometown for a better life	Certainty in control	13
……	…	…

An individual’s accurate perception of self and future is crucial to their mental health (Taylor and Brown, 1988). Studies have indicated that interpersonal security can effectively promote the connection between the individual and the outside world, narrowing the boundaries between the inside and the outside of the group (Zhang et al., 2015). Simultaneously, when an individual lacks a sense of control over the future, anxiety, stress, and depression accompany this. Based on the item collection and primary research, “have like-minded friends” refers to “interpersonal security” and “have a safe environment to ensure the implementation of my plan” refers to “certainty in control.” We summarized these statements about the safety perception of urban residents in relation to their own psychological status as “self-psychological security,” resulting in the development of 6 scale items.

The degree of urban residents’ sense of recognition with the city can directly reflect the urban integration degree of the inflow population. Having a good occupation is an important economic foundation and a spiritual pillar for urban people to live in the city and it is an important way to engage in social interaction and realize the individual’s value. Research has indicated that social security (Foster et al., 2016; Prieto Curiel and Bishop, 2017), food security (Martin et al., 2016; Tseng et al., 2017), and related factors all have an impact on psychological security. We noted that there were a lot of emotional expressions about cities, society, and occupations in the collected statements. We classified them in detail. For example, “this city is my home” and “no matter how fun outside, you still have to come back” refer to “urban belongingness”; “I like my job very much” and “I am worried that I can’t shoulder the pressure of work” refer to “occupational security”; “Afraid of being scammed by the internet” and “may not be well cared when I am old” refer to “social risk perception.” Interestingly, we have found that technological advancement has increased people’s negative psychological pressure while improving their quality of life. These statements refer to the aforementioned phenomenon in the following way: “technology is progressing too fast, it is difficult to adapt” and “it takes a long time to play games every day, sometimes I feel empty.” refer to “technology risk perception.” In consideration of our literature review, residents’ sense of attachment to the living city, occupational stability, social risk perception, and negative perception of technology were summarized as “social environmental security,” resulting in the development of 13 scale items.

In recent years, more and more people are paying increasing attention to the impact of environmental pollution and climate change on their health. Burnett et al. (2018), Chen et al. (2018), and Obradovich et al. (2018) have also found that air pollution and climate change not only threaten people’s lives but also have significant positive impact on mental illness. Some of the statements show the public’s close attention to the state of the environment. For example, “there is often smog, the environment is poor” and “the air is not good, the pollution problem is very serious.” Consequently, we summarized “environmental pollution risk perception,” “climate change risk perception,” and “natural disaster risk perception” as “natural environmental security,” which reflects urban residents’ perceptions of their own surrounding environment, resulting in the development of five scale items.

**FIGURE 2:**
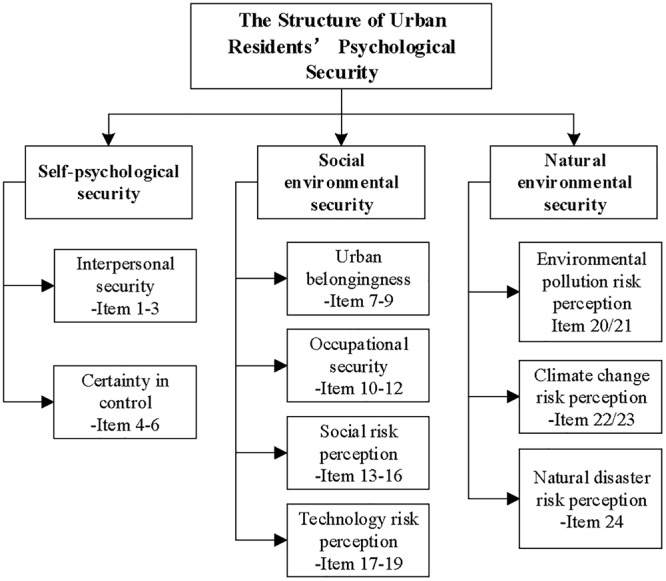
The structure of urban residents’ psychological security.

Based on our review of prior studies and numerous discussions, several experts reorganized, classified, and extracted the expressions to develop a URPS scale that consisted of 24 items. The specific structure is shown in Figure 2. The purpose of this research was to enhance the theoretical logic and content validity of the assessment of urban residents’ psychological security through qualitative research methods. In the next stage, quantitative research methods were used to present and examine the measure through obtaining empirical data.

## Quantitative Method

### Preliminary Survey and Extraction of the URPS Scale

#### Participants

The purpose of this preliminary survey was to evaluate the quality of the initial questionnaire, to purify and correct the items in the initial questionnaire, and to develop the formal URPS scale. In June 2018, we conducted preliminary surveys of residents in different urban areas. Firstly, through haphazard sampling, the research team members publicized and spread the network links of the online questionnaire on social platforms, and expanded the number and scope of the respondents by constantly forwarding links. Secondly, in order to make the distribution of the surveyed population in the demographic characteristics reasonable, stratified random sampling was adopted to distribute some questionnaires with the help of China’s professional questionnaire survey website. Finally, we compared the selected demographics with the national demographics. Survey sample demographics conformed well to the national demographics. Meanwhile, to ensure resident’s active participation, we provided cash rewards after completing the questionnaire.

409 questionnaires were collected. We deleted questionnaires with missing options or more than eight consecutive questions selecting the same option, and identified 304 valid questionnaires (74.3%). We conducted a descriptive statistical analysis of the preliminary survey samples and found that: 47.7% were males and 52.3% were females; the distribution of age was the reflection of the distribution in social reality, with 24.3% of the individuals below the age of 25, 35.2% between 26 and 35, 28.6% between 36 and 45, and 11.8% older than 45. The samples were suitably representative.

#### Procedure

First, we performed a reliability test on the initial scale. (1) Cronbach’s α coefficient was used to judge the overall credibility of the scale. After reverse scoring of the items 1–6, 13–15, and 17–19, the results showed that the Cronbach’s α value of the URPS scale was 0.788, indicating that the overall reliability of the scale was acceptable. (2) Project analysis was used to determine the credibility of every item, including a total of four methods: (1) Descriptive statistical analysis. The descriptive statistical data for each item was used to assess the basic quality of that item, and there were no low-discrimination items with standard deviations less than 0.75. (2) Extreme group test. Among the 304 residents surveyed, we selected 27% of the highest total scores and 27% of lowest total scores, that is, a total of 167 people whose score was higher than 82 points or below 167 points as extreme groups, and we performed independent sample *t*-tests for the extreme groups. The *t*-test values all reached a significance level of 0.05, indicating that all the items can effectively identify the high and low scores. (3) Correlation test. Among the 24 questions in the scale, all the items were significantly correlated with the total score of the scale. (4) Cronbach’s α value test. The data showed that the overall credibility value of the scale would decrease after deleting any item. Thence, after the project analysis, there were still 24 items in the URPS scale.

Second, we conducted principal component analysis on the 24 items. During the analysis, we removed any item with a factor load value less than 0.5 or a cross load value over 0.4. After multiple factor analysis, the 7th, 16th, 17th, and 18th items were deleted, and a well-discriminating factor structure was obtained. Consequently, we developed a URPS scale with 20 items.

Finally, based on the feedback from some interviewees and the re-discussion of experts, we improved the linguistic expression of the scale items, thereby further improving the accuracy and clarity of the scale expression and improving the content validity of the scale.

In summary, we improved the quality of the initial scale through conducting a pre-study assessment and a formal survey using the URPS scale consisting of 20 items. (see Supplementary Appendix). The scale was used in the formal survey.

### Formal Survey and Structural Analysis of the URPS Scale

#### Data Collection

In February 2019, we collected data using questionnaires. A total of 1,036 formal questionnaires was sent out and 985 copies were returned, of which 802 were valid, and the effective recovery rate was 77.4%. The specific distribution of the sample is shown in Table 4.

**TABLE 4:** Sample distribution.

**Sex**	***N***	**Age**	***N***	**Marital status**	***N***
Male	389	<18	1	Married	497
Female	413	18–25	233	Spinsterhood	292

**Nature of organization**	***N***	26–35	252	Else	13
			
Government	22	36–45	215	**Monthly income (RMB)**	***N***
				
Public institution	152	46–55	85	<3000	108
State-owned company	151	>55	16	3000–5000	169
				
Collectively ownership institution	12	**Diploma level**	***N***	5001–8000	225
				
Private company	255	Senior high school and following	40	8001–10000	128
Joint venture company	53	Junior college	96	10001–20000	131
Sino-foreign joint company	12	Bachelor’s degree	561	20001–50000	38
Foreign-funded company	39	Master’s degree	85	>50000	3
Joint-stock company	30	Ph.D. and above	20		
Else	73				

#### Exploratory Factor Analysis

Exploratory factor analysis was performed on the optimized scale using SPSS 19.0 with half of the data (*N* = 401). As the KMO value of the scale was 0.803 > 0.8, the Bartlett test was passed (*p* = 0.000 < 0.001), indicating that the variables correlated and were suitable for factor analysis. The principal component analysis method and varimax orthogonal rotation were used to obtain the factor load matrix as shown in Table 5. According to the Kaiser criterion, we extracted four factors with eigenvalues higher than 1, and the accumulated variance explanation rate of these four factors was 52.5%.

**TABLE 5:** Exploratory factor analysis results.

**Item**	**Commonality**	**Factor**			
		**S1**	**S2**	**S3**	**S4**
CCRP-1	0.688	0.819			
EPRS-2	0.646	0.793			
CCRP-2	0.604	0.771			
NDRP	0.566	0.728			
EPRS-1	0.474	0.678			
IS-1	0.592		0.763		
IS-3	0.590		0.739		
IS-2	0.570		0.736		
CC-1	0.520		0.664		
CC-2	0.465		0.619		
CC-3	0.460		0.558		
SRP-1	0.568			0.744	
SRP-2	0.587			0.720	
SRP-3	0.469			0.653	
TRP	0.418			0.571	
UB-1	0.480				0.691
OS-1	0.530				0.683
OS-3	0.464				0.633
OS-2	0.405				0.608
UB-2	0.403				0.596

**Factor name**		**Natural environmental security**	**Self-psychological security**	**Social environmental risk perception**	**Social security**

Eigenvalues		4.054	3.268	1.881	1.297
Factor variance contribution%		20.269	16.339	9.404	6.486
Accumulated variance contribution%		20.269	36.607	46.012	52.498

*CCRP, climate change risk perception; EPRS, environmental pollution risk perception; NDRP, natural disaster risk perception; IS, interpersonal security; CC, certainty in control; SRP, social risk perception; TRP, technology risk perception; UB, urban belongingness; OS, occupational security.*

Combining the items of each dimension and the analysis of the related literature, we named and defined the four scale factors explored by principal component analysis as follows:

(1) “Natural environmental security” (5 items), is the overall perception of urban personnel on the natural environment state of living cities;
(2) “Self-psychological security” (6 items), is the safety expectation of urban personnel for future life and interpersonal relationships according to their past life experience;
(3) “Social security” (5 items), as the individual’s sense of stability and belonging within urban life.
(4) “Social environmental risk perception” (5 items), is the individual’s overall perception of social risk in urban life.

#### Confirmatory Factor Analysis

We used the other half of the data sample (*N* = 401) to test how well the conceptual model obtained by the exploratory factor analysis fit the actual observed data. In order to better verify the accuracy of the model, four competition models are proposed below, which are compared with the results of the above exploratory factor analysis.

We set Four alternative models:

M1: single factor model in which we hypothesized that the 20 items had a common latent variable: URPS.
M2: two-factor model in which we hypothesized that 11 items from natural environmental security and self-psychological security would have common latent variables, and 9 items from social environmental security have common latent variables.
M3: the three-factor model in which we hypothesized that 5 items of natural environmental security would have common latent variables, 6 items of self-psychological security would have common latent variables, and 9 items of “social security” and “social environmental risk perception” would have common latent variables: social environmental security.
M4: four-factor model in which according to the results of exploratory factor analysis, we hypothesized that the four factors of “social security” and “social risk perception” in natural environment security, self-psychological security, and social environmental security would be factors in this model.

For each of the above models, we used each factor as the latent variable and the corresponding items as the observational variables to perform confirmatory factor analysis, and the model fit results are shown in Table 6. The fit results for M1, M2, and M3 were not ideal. The GFI, AGFI, NFI, CFI, TLI, and IFI for three models were all less than 0.9, and the RMSEA value of M1 and M2 were both greater than 0.1. The *χ*^2^/*df* of the M4 model was 2.009, which is the smallest when compared to the other three models, and the GFI, AGFI, CFI, TLI, and IFI of M4 were all greater than 0.9. Therefore, we considered that the M4 model was the optimal first-order model.

**TABLE 6:** Major fitting degree indices of urban residents’ psychological security.

**Model**	***χ*^2^**	***df***	***χ*^2^/*df***	**GFI**	**AGFI**	**NFI**	**CFI**	**TLI**	**IFI**	**RMSEA**
M1: Single factor model	1525.020	170	8.971	0.618	0.529	0.342	0.363	0.288	0.369	0.141
M2: Two-factor model	1235.130	169	7.308	0.685	0.609	0.467	0.499	0.436	0.503	0.126
M3: Three-factor model	543.978	167	3.257	0.866	0.831	0.765	0.823	0.798	0.825	0.075
M4: Four-factor model	329.439	164	2.009	0.923	0.901	0.858	0.922	0.910	0.923	0.050

However, there were still some indicators that did not meet expectations. We revised the model parameters and released the variance coefficients with a correction index greater than 10, as shown in Table 7.

**TABLE 7:** Overall fitting degree indices of each modification.

		**Initial model fitting**	**Release e16-e17**	**Release e10-e11**	**Assessment**
Absolute fitting index	*X*^2^	329.439, *df* = 164 *P* = 0.000	282.753, *df* = 163 *P* = 0.000	259.410, *df* = 162 *P* = 0.000	Great
	GFI	0.923	0.933	0.938	Great
	RMR	0.065	0.062	0.062	Good
	RMSEA	0.050	0.043	0.039	Great
Relative fitting index	AGFI	0.901	0.914	0.920	Great
	NFI	0.858	0.878	0.888	Good
	TLI	0.910	0.934	0.946	Great
	CFI	0.922	0.944	0.954	Great

**FIGURE 3:**
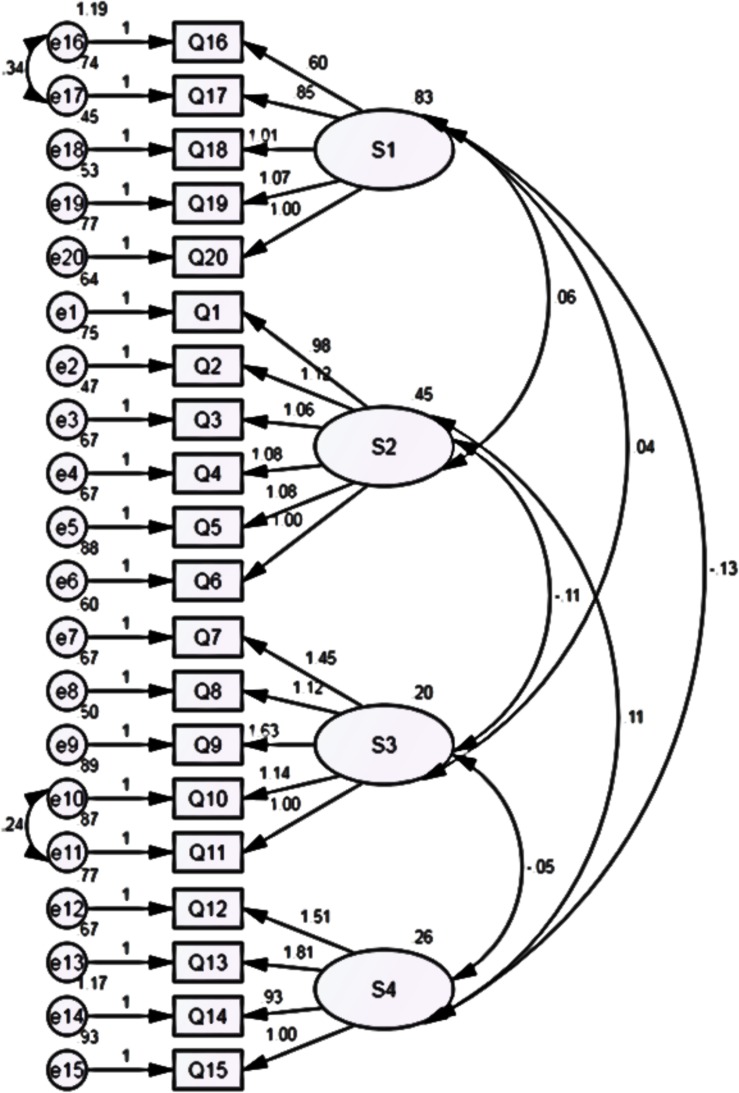
Estimations of the standardized path coefficient of the final confirmatory factor model.

After twice model corrections, the GFI, AGIF, NFI, TLI, and CFI values were all greater than 0.9, the RMSEA value was below 0.05, and the *χ*^2^/*df* value was 1.601, indicating that the data fit well with the model, and all indicators achieved good results. Thus, the URPS model had an ideal fit. The standardized path diagram is provided in Figure 3.

#### Reliability and Validity

The evaluation of the reliability of the scale mainly included two levels of the overall credibility of the scale and the credibility of the latent variables. The Cronbach’s α value (>0.7) was used to test the overall credibility of the scale and the credibility of the latent variable was tested by both the Cronbach’s α value and CR value. The analysis showed that the overall Cronbach’s α value of the URPS scale was 0.773, indicating that the overall credibility of the scale is reliable. The CR value of each latent variable was between 0.75 and 0.9, and the Cronbach’s α values for each latent variable were 0.828, 0.806, 0.686, and 0.670, respectively. Since each principal component is not measured as a single variable and has fewer items, the reliability values were within acceptable limits and the scale passed the reliability test.

The evaluation of the validity of the scale mainly included two aspects: content validity and structural validity. The content validity was ascertained using qualitative methods. The verification of structural validity examines the convergence validity and discriminant validity of the scale. We strictly followed standard scale development procedures. We conducted a large scale literature review, collected initial items through in-depth interviews based on grounded theory, invited management experts to discuss the design of the questionnaire repeatedly, and a pre-study utilizing 304 questionnaires, so the content validity of this scale is reliable. In addition, the standardized load of 20 scale items at the corresponding latent variables was greater than 0.5 and reached the level of statistical significance, and the corresponding AVE value was between 0.45 and 0.65, which satisfies AVE > 045, indicating good convergence validity of the scale. The square root of the AVE of the latent variable was greater than the correlation coefficient between the latent variables, indicating that the potential structural discrimination of the variable was better. The scale passed the validity test. The specific analysis is shown in Table 8.

**TABLE 8:** Reliability and validity test of latent variables.

	**Natural environmental security**	**Self-psychological security**	**Social environmental risk perception**	**Social security**
Natural environmental security	0.796^∗^			
Self-psychological security	0.391	0.740^∗^		
Social environmental risk perception	–0.066	0.330	0.679^∗^	
Social security	0.371	–0.458	–0.052	0.711^∗^
**Cronbach’s α**	0.828	0.806	0.686	0.670
**CR**	0.8733	0.8493	0.7914	0.7762
**AVE**	0.6343	0.5472	0.4606	0.5060

*^∗^indicates the square root of AVE value.*

#### Criterion Correlation Validity

We used the psychological security of urban residents measured by single global rating as the criterion. Respondents answered one question about their general feeling of security in urban life: “Based on your daily life in the city, what do you think your psychological security score is?” The question was scored on a Likert scale, in which 1 means “very unsafe,” and 5 means “very safe.”

Harman single factor test was carried out on 21 items including the URPS scale and the item of single global rating. The results showed that 21 items were automatically divided into 4 factors instead of one factor, and the variance contribution rate of the first main factor was 19.706%, which was much less than 40%. It can be seen that the common method bias has no significant interference with the criterion correlation validity test.

As shown in Table 9, there was a significant positive correlation between the mean value of the URPS scale and the results measured by single global rating. The four main factors scores of the scale were also significantly correlated with the score of psychological security, with a correlation coefficient between 0.2 and 0.4. To further investigate the explanatory power of the scale regarding psychological security, we conducted regression analysis. First, gender, age, education background and income as demographic variables were used as variables in model 1, and the adjusted *R*^2^ was only 0.023, thus indicating that demographic variables explained only 2.3% of psychological security. Then, four main factors were included in model 2, and the adjusted *R*^2^ was 0.219, and the *F* value was significant at the 0.001 level, thus indicating that the four factors of the scale had a significant positive prediction effect on psychological security. Finally, the mean value of the scale was included in model 3, and the adjusted *R*^2^ was 0.191, and the *F* value was significant at 0.001, thus indicating that the mean value of the scale was able to significantly positively predict the psychological security of urban residents. Therefore, the URPS scale developed in this paper had good criterion correlation validity.

**TABLE 9:** Correlation coefficient and regression results.

**Variable**	**Natural environmental security**	**Self-psychological security**	**Social environmental risk perception**	**Social security**	**Mean of URPS**
Psychological security	0.363^∗∗∗^	0.213^∗∗∗^	0.261^∗∗∗^	0.363^∗∗∗^	0.427^∗∗∗^

		**Psychological security**				
		
		**Model 1**	**Model 2**		**Model 3**

Constant		3.382^∗∗∗^	1.490^∗∗∗^		1.699^∗∗∗^
Gender		−0.108^∗^	–0.117^∗∗^		−0.112^∗^
Age		–0.011	–0.016		–0.014
Education		0.085^∗^	0.08^∗^		0.080^∗^
Income		0.045^∗^	–0.016		0.001
Natural environmental security			0.174^∗∗∗^		
Self-psychological security			0.117^∗∗∗^	
Social environmental risk perception			0.311^∗∗∗^	
Social security			0.051^∗^	
Mean of URPS					0.594^∗∗∗^
*F*		5.801^∗∗∗^	29.042^∗∗∗^		38.859^∗∗∗^
*R*^2^		0.028	0.227		0.196
Δ*R*^2^		0.023	0.219		0.191

*^∗^*p*<0.05; ^∗∗^*p*<0.01; ^∗∗∗^*p*<0.001.*

## Discussion and Conclusion

### Discussion

We attempted to integrate the conceptual connotation of URPS by borrowing the elements from diverse literature. A scale comprising three dimensions (psychology, society and environment) was developed. The measurement of URPS from the dimension of self-psychological security, natural environmental security and social environmental security has objective rationality, thus authentically and explicitly demonstrating the current state of URPS. For example, Zhang (2007) have divided the feeling of security of residents into psychological security, social security, economic security government security and environmental security. However, Zhang did not consider the influence of climate change risk perception, technology risk perception, urban belongingness and other factors. Moreover, although the survey was conducted in China, the scale is not only suitable for developing countries that have achieved rapid economic growth at the expense of the environment, such as China and India, but also is suitable for developed countries that have strict environmental requirements, such the European Union and the United States.

The dimension of self-psychological security was established on the basis of previous studies, including interpersonal security and certainty in control. Gunn et al. (2014) have found that interpersonal distress decreases people’s sense of security, in agreement with the results of this paper. People who cannot trust others and who avoid others as much as possible in interpersonal communication cannot accept themselves well and tend to make negative comments about themselves, thereby affecting their psychological security (Cong and An, 2004). Steptoe et al. (2007) and Chou and Chi (2001) believe that a low sense of control is associated with depressive symptoms, thus supporting the factor of certainty in control in this paper. People with a lower sense of control often feel that their lives are out of control or a mess, or that they cannot cope with life’s unexpected problems; consequently, they are always in a state of insecurity. Therefore, we believe that interpersonal security and certainty in control can effectively reflect the state of URPS.

The dimension of natural environmental security includes air pollution risk perception, climate change risk perception and natural disaster risk perception. To date, pollution and climate change in environmental factors have rarely been considered in the development of the psychological security scale of urban residents; this consideration can be regarded as an innovation of this paper. Jacquemin et al. (2007), Lucchini et al. (2012), and Sucker et al. (2008) have found that exposure to pollution stimulates nerves in the brain, thus causing negative emotions such as worry, anxiety, tension and aggression. Having negative emotions for a long time increase individuals’ sense of dissatisfaction and vigilance, and affects their sense of security. Although there is still controversy in the public opinion on whether global climate change exists and whether it can threaten human life (Leiserowitz, 2005; Weber and Stern, 2011), the risk perception of extreme cold and hot weather, sea level rise and food loss brought by climate change, are real threats to people’s psychological security. If an individual has experienced natural disasters such as tsunamis, earthquakes, floods or tornadoes, a trauma will result that is difficult to heal for individual psychology (Weinstein et al., 2000; Williams, 2006). People who have experienced trauma show severe stress reactions over a long period. They are extremely sensitive to external threats and may have long-term mental disorders that severely affect their psychological security. Therefore, urban residents’ perception of the risks of air pollution, climate change and natural disasters play a key role in the URPS.

The dimension of social environmental security includes two factors: social security and social risk perception. Social security includes urban belongingness and occupational security. Social risk perception includes medical, pension, food and technology risk perception. The factor of urban belongingness is the reflection of psychological security in the urban context; consideration of this factor is another unique feature of this paper, as compared with the general psychological security scale. The sense of city identity increases residents’ living satisfaction and brings about positive psychological expectations (Zenker and Petersen, 2014). The economic factor is the guarantee of individual security, and the main economic security of urban residents is based on having a stable occupation. Whether the city is able to provide satisfactory jobs is a key issue for urban residents (Vieitez et al., 2001), and also are the main factors in this paper. Moreover, Bodie et al. (2009), Hesketh et al. (2012), Yan (2012), Gille et al. (2015), and Wu et al. (2017) believe that medical supervision, pension resources, food safety and other issues have caused urban residents to have negative emotions, such as anxiety. Therefore, urban belongingness, occupational status and social factors can directly influence the psychological security of urban residents.

In addition, we also found that the negative spillover effects brought about by the development of technology affect the individual’s mental health. This can be considered as a new development in the field of psychological security structures of urban residents. Most previous research has focused on the benefits of technological advances, such as general increases in productivity and quality of life. Internet technology is widely used worldwide and can connect people across distances and enhance interpersonal communication, such as cross-border communication. However, we found in the interviews that the rapid updating of technology makes elderly people or those with low adaptability fear being abandoned by the times, and their unfamiliarity with the Internet leads to their fear of being swindled and robbed. Young people are more familiar with the online environment, but they spend too much time communicating on the Internet and thus neglect the real world. Moody (2001) as found that the massive use of Internet technology has caused some people to be lonely and socially isolated in the real world, in agreement with our findings from this study. Some researchers believe that lonely individuals use the Internet more to modulate negative moods and obtain emotional support (Morahan-Martin and Schumacher, 2003). This paper argues that individuals too immersed in the semi-virtual world of the Internet will expend a large amount of emotional energy, leading to emotional exhaustion and interpersonal alienation in the real world. Excessive feelings of loneliness and alienation reduce the individual’s psychological security.

### Conclusion

(1) We first conducted in-depth interviews with 25 urban residents, and combined with a literature review, developed the initial URPS scale consisting of 24 items through qualitative analysis. Subsequently, we used project analysis and principal component analysis to purify the scale and verify the structure of scale, using 304 pre-survey questionnaires, and then developed a formal survey URPS scale, with 20 items.
(2) A total of 802 formal questionnaires were collected. Through principal component analysis of 401 samples, “natural environmental security,” “self-psychological security” and “social environmental security” (including social security and social risk perception) were obtained. The KMO value was 0.803, which is greater than 0.7, the significance was 0.000, and the cumulative variance of the four factors was 52.498%. We performed confirmatory factor analysis on the other half of the data and found that the M4 model was superior to the other three models. Simultaneously, because some indicators were not excellent, the model parameters were corrected. The GFI, AGIF, TLI and CFI values of the modified model were 0.938, 0.920, 0.946, 0.954, respectively. The RMSEA value was 0.039, and the *χ*^2^/*df* value was 1.601. In summary, the good range showed that the URPS model had an ideal fit.
(3) Reliability test and validity test were performed on the developed scale. Cronbach’s α value of the overall credibility of the scale was 0.773, which is higher than 0.7, and Cronbach’s α values for each latent variable were 0.828, 0.806, 0.686, and 0.670, respectively. The CR values for each latent variable were 0.8733, 0.8493, 0.7914, and 0.7762, separately. On the basis of accepted standards, the scale passed the reliability test. The scale was developed in strict accordance with recommended procedures and the development was scientific and rigorous. Analyses demonstrated that the content validity was reliable. The standardized loads of 20 scale items at the corresponding latent variables were all greater than 0.5, and the corresponding AVE values were 0.6343, 0.5472, 0.4606, and 0.5060, respectively, all of which were above 0.45. The scale convergence validity was high, and the square root of the AVE of the latent variable was greater than the correlation coefficient between the latent variables. In addition, the degree of potential variable structural discrimination was better. Importantly, the scale also passed the validity test. In the criterion correlation validity test, the correlation coefficient between the results of psychological security of urban residents measured by single global rating and the mean of the URPS scale is 0.427, and the correlation coefficients between it and the mean of each dimension were 0.363, 0.213, 0.261, and 0.363, respectively. Regression analysis showed that URPS scale was able to significantly predict psychological security at the 0.001 level, with ideal criterion validity.

## Limitations and Future Studies

There are some limitations in this study: (1) there are regional limitations in the choice of samples. Although the samples used were representative of most demographic variables when taking into account the economically developed and underdeveloped regions of China but there are still some areas that were not involved in this study, and there is no distinction between scales for different levels of urban development. (2) The focus of the research was on urban residents, so a large number of rural residents who complete the questionnaires were deleted, and this led to the lack of a comparative analysis between rural and urban residents. (3) The main contribution of this study was to develop a psychological security scale for urban residents, which has not been empirically tested. Therefore, it is necessary for the scale to be further verified, revised, and improved upon in future research.

Owing to the limitations of the development site, the validity of the scale was verified only in China. We expect to use this scale to measure and compare the psychological security of urban residents in different countries and cities in the future, and to verify that the URPS scale is applicable to different countries and regions. Next, we will conduct a large sample investigation by using the URPS scale. Then, we will analyze the differences in dimensions/variables among different regions and determine whether economic development, environmental pollution and technological development of different regions have significant differences in the four major factors, on the basis of the sample data. At the same time, urban residents’ psychological security can be used as a mediator to study the resident turnover rate, sense of city integration and urban crime rate to improve city management level and city attraction.

## Data Availability Statement

All datasets generated for this study are included in the article/Supplementary Material.

## Ethics Statement

This study was carried out in accordance with the principles of the Basel Declaration and recommendations of Ethical Codes of Consulting and Clinical Psychology of Chinese Psychological Society, Chinese Psychological Society. The protocol was approved by the Ethics Committee at the Department of Organizational and Behavioral Sciences, China University of Mining and Technology. All subjects gave written informed consent in accordance with the Declaration of Helsinki. Before the interview, the interviewees were told that they would be recorded and that we would fully respect their wishes.

## Author Contributions

JW analyzed the data and wrote the manuscript. RL designed the framework of this manuscript. HC obtained the data and provided suggestions for improvement. QL made a major contribution to the manuscript revision process.

## Conflict of Interest

The authors declare that the research was conducted in the absence of any commercial or financial relationships that could be construed as a potential conflict of interest.
