# Supplementary material for: Measuring the Psychological Security of Urban Residents: Construction and Validation of a New Scale
Source: Front Psychol. 2019 Oct 25;10:2423. doi: 10.3389/fpsyg.2019.02423 (PMC6824293; doi:10.3389/fpsyg.2019.02423)
Supplement: Supplementary file 1 [file Presentation_1.pdf]

Appendix: URPS Scale

1. The people around me think that I am a shy and introvert.
2. I am afraid to establish and maintain close relationships with others.
3. I never dare to take the initiative to say what I think.
4. I always worry that my life will become a mess.
5. I often worry I will lose control of my thoughts or emotions.
6. I feel that life is always full of uncertainty and unpredictability.
7. I think my city is perfect and can meet all my needs.
8. My city is economically developed or the economy is developing rapidly.
9. In the city where I live, I can find a job that I am satisfied with.
10. I have a stable job and I don't worry about unemployment unless I voluntarily resign.
11. My work is almost no danger, and I can afford the current amount of tasks.
12. I am very worried about medical problems for myself or my family (such as medical expenses and medical standards).
13. I am more worried about the issue of old-age care, and the social pension system is still not perfect.
14. I am worried about health issues when buying food or eating out.
15. Although social networks are getting more developed, I always feel that people are more alienated.
16. I am not worried about the harm of air pollution such as haze to me.
17. I am not worried about the problem of water pollution.
18. I am not worried about extreme climate change (such as severe summer heat and severe winter cold) caused by the greenhouse effect.
19. I am not worried about the rise in sea level caused by climate change.
20. I am not worried about natural disasters such as earthquakes, tsunamis and typhoons.
